# Effects of Berberine and Its Derivatives on Cancer: A Systems Pharmacology Review

**DOI:** 10.3389/fphar.2019.01461

**Published:** 2020-01-15

**Authors:** Chaohe Zhang, Jiyao Sheng, Guangquan Li, Lihong Zhao, Yicun Wang, Wei Yang, Xiaoxiao Yao, Lihuan Sun, Zhuo Zhang, Ranji Cui

**Affiliations:** ^1^ Jilin Provincial Key Laboratory on Molecular and Chemical Genetic, Second Hospital of Jilin University, Changchun, China; ^2^ China-Japan Union Hospital of Jilin University, Changchun, China

**Keywords:** berberine, tumor, systems pharmacology, liver cancer, breast cancer

## Abstract

Numerous studies have shown that berberine and its derivatives demonstrate important anti-tumor effects. However, the specific underlying mechanism remains unclear. Therefore, based on systems pharmacology, this review summarizes the information available on the anti-tumor effects and mechanism of berberine and its derivatives. The action and potential mechanism of action of berberine and its derivatives when used in the treatment of complex cancers are systematically examined at the molecular, cellular, and organismic levels. It is concluded that, with further in-depth investigations on their toxicity and efficacy, berberine and its derivatives have the potential for use as drugs in cancer therapy, offering improved clinical efficacy and safety.

## Introduction

Berberine (chemical formula: C_20_H_18_NO_4_, slowly soluble in water), the main alkaloid in the herbal medicine coptis, has been widely used in China (Alolga et al., 2016; Li et al., 2019). It also exists in many Berberidaceae, Papaveraceae, and Rutaceae plants. Based on its chemical structure, berberine is a protozoan morphinane alkaloid (Ruan et al., 2017). In addition to extensive biological activities, such as anti-inflammation, antioxidative, and anti-diabetic, berberine demonstrates anti-tumor activity by means of interference in tumorigenesis and in multiple features of tumor development. Therefore, berberine is widely used in the prevention and treatment of tumors (Refaat et al., 2015; Li et al., 2017a; Liao et al., 2018; Liu et al., 2019a). In addition, it is difficult for berberine to penetrate cytomembrane and to be assimilate into the gastrointestinal tract due to its poor lipid solubility (Lu et al., 2006; Ge et al., 2016; Zhu et al., 2017). Therefore, a series of berberine derivatives have been designed by transforming and modifying its chemical structure (Sagar et al., 2006; Xiao et al., 2018). Simultaneously, the pharmacological activities of berberine derivatives were also widely studied.

**Figure 1:**
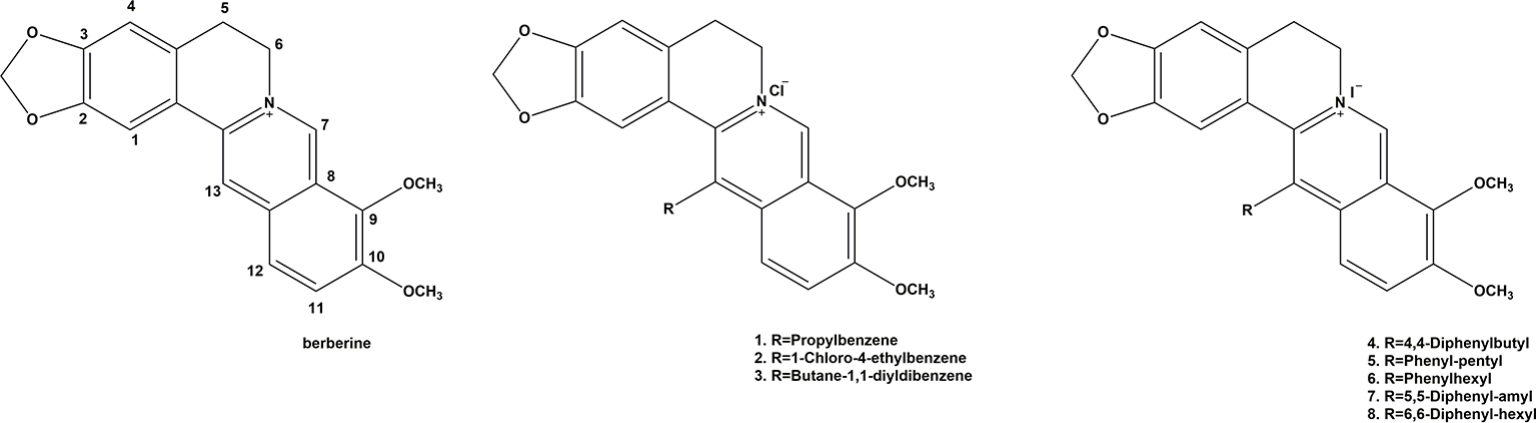
Possible mechanism of the anti-tumor effects induced by berberine based on systems pharmacology. VEGFR, vascular endothelial growth factor receptor; Akt, serine/threonine kinase; ERK, extracellular regulated protein kinases activation; MMP, Matrix Metalloproteinase; AMPK, AMP-activated protein kinase; BAX, Bcl-2-associated X protein; Bcl-2, B-cell lymphoma 2; ROS, reactive oxygen species.

Numerous studies have revealed that berberine has anti-tumor activity in many cancers (Wen et al., 2016; Zou et al., 2017). This mainly consists of inhibiting tumor cell proliferation and tumor angiogenesis, inducing apoptosis of tumor cells, and delaying the transfer of tumor cells (Li et al., 2018; Dai et al., 2019; Liu et al., 2019b). In contrast, in a leukemia mouse model, berberine reportedly inhibited the growth of the spleen by inhibiting the differentiation of nicotinate mononucleotide pyrophosphorylase and granulocyte (Yu et al., 2007; Gu et al., 2008; Wang Z. et al., 2016). In the SCC-4 tumor mouse model, the number of tumors in mice treated with berberine was obviously less than that observed in the control group (Jantova et al., 2003; Yu et al., 2007; Ho et al., 2009). In this review, we focus on the anti-cancer mechanisms and signaling pathways of berberine (**Figure 1**).

## Effects of Berberine on p53 and the Cell Cycle

Berberine alters cellular processes through interactions with nucleic acids and various proteins. The effect of berberine on cell cycle progression has also been observed through cell cycle arrest at the G1 phase, G0/G1 phase, or G2/M phase (Eo and Kim, 2014; Gu et al., 2015). For example, berberine induced G2/M phase arrest in T47D and in G0/G1 in MCF-7 cells (Barzegar et al., 2015). Computational and experimental data demonstrated that CaM potentially plays a crucial role in the anti-tumor effect induced by berberine. A biological assay revealed that berberine induced G1 cell cycle arrest in Bel7402 cells partially by interacting with CaM and blocking subsequent signal cascades (Ma et al., 2013).

p53 is one of the key tumor suppressor genes and is very important in the process of apoptosis in various tumor cells. The protein encoded by this gene is a transcriptional factor that controls the initiation of the cell cycle. Therefore, p53 plays a critical role in whether to start cell division or not. If the cell is damaged and cannot be repaired, p53 protein will participate in the initiation process, allowing the cell to initiate apoptosis. For example, p53 can inhibit BCL2 by BAX, which increases the BAX/BCL2 ratio and can also induce cell apoptosis by Apaf-1 regulation of the underlying signal caspase 3 (Babiker et al., 2018; Liu et al., 2018; Ma et al., 2019; Nkpaa et al., 2019). The tumor suppressor p53 was reported to play a key role in the anti-tumor action of berberine. A recent study has revealed that berberine may up-regulate p53 expression by suppressing the inner inhibitor MDM2 at the post-transcriptional level (Shukla et al., 2016; Wang et al., 2016; Chrysovergis et al., 2019; Draganov et al., 2019). In addition, berberine may increase the expression of the primary precursor, precursor, and mature forms of miR- 23a, which could enhance berberine-induced G2/M cell cycle arrest. Orally administered berberine inhibits p53 expression and non-expression in lung cancer xenografts, increasing the level of p53 and therefore inhibiting the G1 phase of tumor cells. CDK (Cyclin-Dependent Kinase) is a heterodimeric protein that promotes the progression of the cell cycle by modulating the kinase cascade. Inhibition of cell cycle G1 is also observed when CDK inhibitors are over-expressed. Berberine induces the over-expression of CIP1/p21 and Kip1/p27 proteins and downregulates cyclin-dependent kinases (cdk2, cdk4, cdk6), leading to G1 arrest of tumor cells (Eom et al., 2008; Lan et al., 2014; Li et al., 2017b).

An interesting study has shown that berberine exerts different effects on p53 expression in breast cancer cell lines MCF-7 and MDA-MB231. MCF-7 cells express the wild-type tumor protein p53 (TP53), while MDA-MB-231 cells express the mutant TP53. In both cell lines, p53 mRNA levels are down-regulated by TPA (12-O-tetradecano-13-acetate, a tumor promoter) (Kim and Jung, 2012). After coptis, the level of p53 expression increased in TPA-induced MCF-7 cells. However, the expression of p53 remained unaltered in TPA-treated MDA-MB231 cells (Yan and Asmah, 2017). Studies have demonstrated that berberine does not directly affect the expression of p53 when p53 is mutated and mediates p53-dependent inhibition of tumor cells in the G2 phase. Therefore, berberine can affect the mutation of p53 and non-mutant p53 in cancer cells through different pathways.

Berberine has been seen to activate TP53, which increased the expression of miR-23a in HCC. This TP53, in turn, stimulated p21Cip1 and GADD45alpha expression. Suppression of miR-23a blocked the binding of TP53 to the chromatin and blocked transcriptional activation of p21Cip1 and GADD45alpha (Wang et al., 2014b; Sujitha et al., 2018). Berberine induced miR-23a, which may not be suppressed in mitosis A (NIMA) kinase 6 (NEK6) and resulted in blocking of the cell cycle in G2/M (Jee et al., 2010). In addition, NEK6 may have an impact on TP53. NEK6 antagonized TP53 and induced senescence (Sheng et al., 2015). RNA-Seq analysis revealed that berberine modified the expression of genes in the TP53 and cell cycle pathways (Li et al., 2015).

TP53 plays an important role in the induction of tumor cell apoptosis. In another study, the effects of berberine on the arachidonic acid (AA) metabolic pathway in HCC were examined. Berberine altered the viability and apoptosis of HCC cells in a dose-dependent fashion by inducing the translocation of apoptosis-inducing factors between the mitochondria and nucleus. Berberine also suppressed the levels of cytosolic phospholipase A2 (cPLA) and COX-2, which increased the ratio of AA to PEG-2 (Feng et al., 2012). Collectively, these findings suggest that tumor protein p53 (TP53) is closely associated with the anti-tumor effects induced by berberine (**Figure 1** and **Table 1**).

**Table 1:** Effects of berberine on various cancer cell lines.

Cell lines	Origin	Effects
MCF-7, MDA-MB-231	Breast cancer	Increased BAX/BCL2 ratio and ROS;
		Decreased VEGFR, Akt, ERK1,2 activation and the expression of MMP-2,9, IL-8;
		Cycle arrest and cell apoptosis
HCC, MHCC97-L, HepG2, SMMC-7721,	Liver cancer	Down-regulation of HNF4alpha and Exo-70, COX-2, NF-kappaB, MMP-
Be-l7402		9, TNF-α, CD147; MAPK and ERK1,2 inactivation;
		TP53, mTORC1 inhibition;
		Cell cycle arrest
HTB-94	Chondrosarcoma	Increased TP53 and p21^Cip1^ expression;
		Decreased cyclin B1, CDC2, CDC25c, and pRB expression.
A549, H1299	Lung cancer	Caspase-3 activation;
		Decrease in Bcl-2/Bcl-xL levels;
		Increase in Bax/Bak levels
LNCaP, PC-3	Prostate carcinoma	Caspase-9 and -3 activation;
		Decrease in Bcl-2/Bcl-xL levels;
		Up-regulation of p21 and p27

VEGFR, vascular endothelial growth factor receptor; Akt, serine/threonine kinase; ERK, extracellular regulated protein kinases activation; AMPK, AMP-activated protein kinase; BAX, Bcl-2-associated X protein; Bcl-2, B-cell lymphoma 2.

## Effects of Berberine on Tumor Proliferation and Apoptosis

Apoptosis is a multi-gene, strictly controlled process. These genes are highly conserved among species, including the Bcl-2 family and caspase family. Berberine may down-regulate the expression of XIAP, an X-linked inhibitor of apoptotic proteins, triggering apoptosis in leukocyte-depleted p53 genes (Liu et al., 2013). Berberine can also induce apoptosis by increasing reactive oxygen species (ROS) in certain breast cancer cells (MCF-7 and MDA-MBA-231). Berberine and tumor necrosis factor-related apoptosis-inducing ligand (TRAIL) demonstrated synergistic effects on apoptosis-induction in TNBC, with p38 MAPK activated in response to the combined treatment. Hence, berberine/TRAIL induced apoptosis by regulating p38 MAPK pathways (Refaat et al., 2015).

Several studies have reported the anti-tumor effects of berberine in the human hepatocellular carcinoma (HCC) cell line by inducing apoptosis. Berberine activates mitochondrial apoptosis in HCC cells by increasing Bax expression, PT pore formation, Cyto C release into the cytosol, and the subsequent activation of caspase 3- and 9-signaling pathways (Wang et al., 2010). CD147 was shown to up-regulate the expression of classic MDR-related transporter MDR1, and it affects apoptotic pathways in cancer cells, enhancing drug sensitivity. CD147 is highly expressed in HCC cells, promoting tumor invasion, metastasis, and tumor angiogenesis, and also inhibiting apoptosis and anoikis (Kuang et al., 2009). Furthermore, Hou et al. (2011) reported that berberine induces both apoptosis and cell death in HepG2 cells, which correlates with the down-regulation of CD147. Hepatic nuclear factor 4 alpha (HNF4alpha), a key liver transcription factor, can transactivate the Exo 70 promoter region. Berberine-mediated cell cycle arrest occurred through the down-regulation of HNF4alpha and Exo-70 (Yu et al., 2014). This demonstrates that berberine induces pyroptosis in HCC. Pyroptosis is a caspase-1 dependent programmed cell death program (Chen et al., 2016). In addition, MiR-22-3p was lower in HCC. Berberine demonstrated an ability to increase miR-22-3p in HCC. In these studies, high doses of berberine inhibited cell growth at the 24 h time interval. Berberine treatment decreased the expression of SP1, cyclin D1, and BCL2. Berberine induced miR-22-3p, which bound SP1 and suppressed cyclinD1 and BCL2.

Berberine has exhibited the ability to overcome multidrug resistance, indicating its potential in tumor chemotherapy. Coadministration of berberine and cisplatin resulted in potentiation, and berberine sensitized the cells to cisplatin. Furthermore, berberine increased the extent of DNA damage and apoptosis normally induced by cisplatin (Zhao et al., 2016). Berberine decreased breast cancer cell migration and chemokine expression. This was determined by wound healing assays and RNA analysis of chemokine receptors in MCF-7 breast cancer cells (Ahmadiankia et al., 2016). Berberine has been proven to increase the anti-tumor effects of tamoxifen (TAM) in drug-sensitive MCF-7 and drug-resistant MCF-7/TAM cells. The combined treatment of berberine and TAM enhanced cytotoxic activity and induced G1 arrest and apoptosis, potentially due to p21Cip-1 induction and increase of the BAX/BCL2 ratio (Wen et al., 2016). The combination of berberine and curcumin has been shown to effectively suppress growth in certain breast cancer cell lines. The combined treatment was more effective than treatment with either berberine or curcumin alone. The combined treatment resulted in phosphorylation of c-Jun N-terminal kinase (JNK) and Beclin1 and reduced the phosphorylation of BCL-2 (Wang K. et al., 2016).

AMPK, an AMP-dependent protein kinase, is a critical molecule in the regulation of bioenergetic metabolism and core metabolic-related diseases. Its activation is accompanied by an apoptotic effect in a caspase-dependent manner *via* the mitochondrial pathway. Activation of AMPK leads to the induction of apoptosis in various human cancer cell types (Ji et al., 2010). Furthermore, berberine promoted AMPK phosphorylation and inhibited Akt phosphorylation in HepG2 cells, leading to caspase-dependent mitochondrial pathway apoptosis (Yang and Huang, 2013). Synergistic antitumor effects were observed when berberine was employed in combination with other agents to treat hepatomas. The combined use of berberine and evodiamine could significantly enhance the apoptosis of SMMC-7721 cells, which is related to the up-regulation of TNF-α (Wang et al., 2008). In addition, the use of berberine in combination with the microtubule poison vincristine has proved efficient against hepatoma cell lines by potentiating the pro-apoptotic effect of the individual drug (Wang et al., 2014a). Other studies have demonstrated that the interstitial implantation of radioactive seed ^125^I induced hepatoma cell apoptosis. This effect was enhanced when ^125^I was combined with berberine, which induces apoptosis, cell degeneration, and necrosis (Wang et al., 2012). Furthermore, the anti-tumor activity of gamma radiation is significantly enhanced by berberine *via* the activation of the p38 MAPK pathway and ROS generation in human hepatoma cells (Ma et al., 2013). Berberine can induce apoptosis and autophagic cell death in HEP-G2 HCC cells. Induction of apoptosis and autophagy require AMP-activated protein kinase (AMPK), resulting in the elevated expression of inactive acetyl-CoA carboxylase (ACC). Inhibition of AMPK by RNAi or the AMPK inhibitor (compound C) suppressed the effects of berberine. In contrast, the AMPK activator AICAR stimulated cytotoxic effects. It has been shown that berberine inhibits mTORC1 activation by stimulating AMPK (Choi et al., 2009). Therefore, these findings suggest that berberine alone or in combination with other drugs possesses an anti-tumor effect mediated *via* AMPK activation.

## Effects of Berberine on Tumor Metastasis Inhibition

Berberine has exhibited its ability to suppress tumor metastasis (Lin et al., 2006; Serafim et al., 2008; Cai et al., 2014). Matrix metalloproteinases (MMPs) degrade the tissue matrix, allowing tumor cells to break through the normal tissue barrier and invade the surrounding normal tissue and distant organs. *In vitro* studies have demonstrated that the inhibition of FAK, IKK, NF-kB, u-PA, MMP-2, and MMP-9 significantly reduced metastasis. Berberine inhibits the release of MMP-2 from tumor cells and thus inhibits tumor cell destruction of the tissue matrix.

Berberine increased the activities of numerous proteins involved in proliferation, such as Janus Kinase 2 (JAK2), Phosphoinositide 3-kinase (PI3K), activator protein-1 (AP-1), and NF-kappaB (Mahata et al., 2011; Fu et al., 2013; Wu et al., 2013; Belanova et al., 2019; El-Zeftawy et al., 2019; Jiang et al., 2019). These proteins decreased IL-8 expression in the TNBC cell line, MDA-MB-231. The IL-8 stimulated invasion was also suppressed by berberine (Kim et al., 2018). Berberine also decreased MMP-2, MMP-9, E-cadherin, EGF, bFGF, and fibronectin in the breast cancer cells. The effect of berberine was inhibited by JNK and p38 MAPK inhibitors and was increased by p38 MAPK activators (Zheng et al., 2014; Zhou et al., 2015; Zhao et al., 2019).

Berberine can also bind to the vasodilator-stimulated phosphoprotein (VASP). VASP is over-expressed in breast cancer cells with high mobility and inhibits polymerization. Berberine binds VASP in MDA-MB-231 cells and suppresses proliferation and tumor growth (Su et al., 2016).

## Structural Modification of Berberine

### Modification Transformation and Antineoplastic Activity of C-13-Substituted Berberine Derivatives

**Figure 2:**
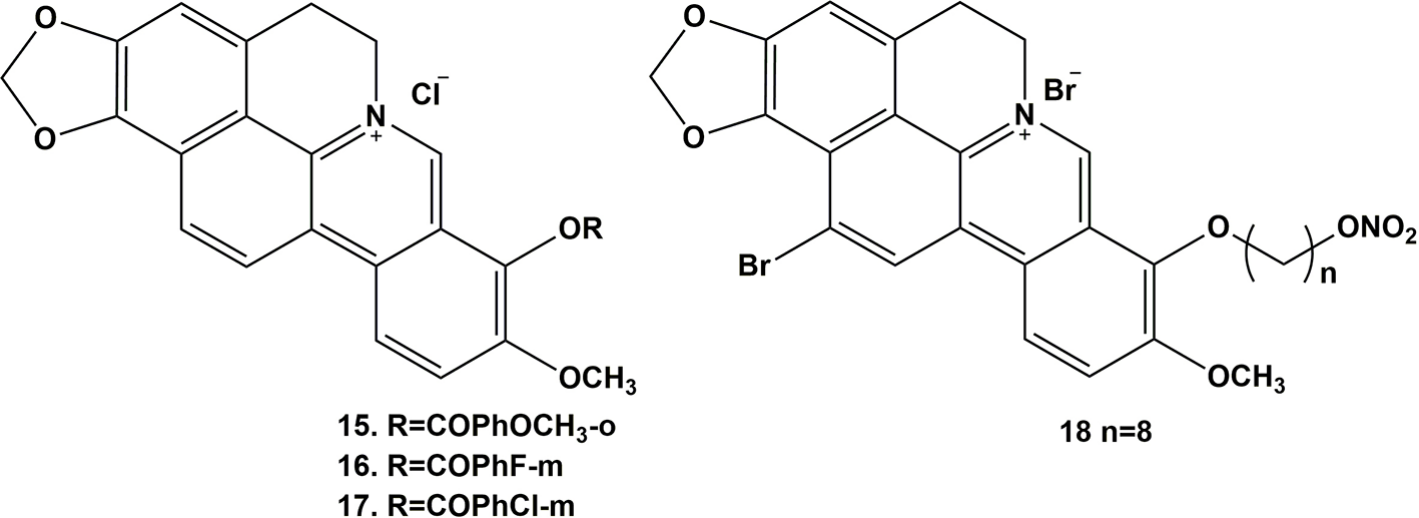
Structure of berberine and compounds 1-8.

The diverse pharmacological properties exhibited by berberine indicate that the alkaloid has definite potential as a drug in a wide spectrum of clinical applications. The structure of berberine (**Figure 2**) represents a biologically essential skeleton and also a natural lead compound for the introduction of various chemical modifications at appropriate positions. The structural modification of berberine for antineoplastic activity has mainly focused on C-9 (Iwasa et al., 1996; Krishnan and Bastow, 2000; Pang et al., 2005; Pang et al., 2007; Cui et al., 2010; Huang et al., 2010) and C-13 (Park et al., 2006; Ortiz et al., 2014). Therefore, to examine the anticancer activity of the berberine derivatives, three berberine derivatives were prepared and bioassayed on human colon carcinoma cell lines. The results revealed that the derivatives also induced cell cycle arrest and cell death by apoptosis. Furthermore, the effect of the derivatives was more potent than that of the parent compound.

To further improve the efficacy and bioavailability of the derivatives, Yang et al. designed several cycloberberine derivatives (Yang et al., 2008; Jin et al., 2015; Yang et al., 2019). Among them, some compounds showed strong inhibition on human HepG2 cells. Another researcher also synthesized a series of cycloberberine derivatives and evaluated their anti-cancer activity (Franceschin et al., 2006). Among them, five compounds exhibited strong inhibition on human HepG2 cells, respectively.

In addition, 13-ethylpyridine hydrochloride berberine derivatives produced by the Franceschin group selectively combined with the G-quadruplex DNA in the cell growth cycle and inhibited the activity of telomerase, demonstrating good anti-tumor activity (Gornall et al., 2007). They also observed that this kind of compound not only increased the stability of G-quadruplex DNA but can also inhibit polymerases (Jin et al., 2015). Gornall et al. studied the interaction between 13-indolyl supersede berberine and G-quadruplex DNA and found that the former can selectively combine with G-quadruplex DNA but not with double-strand DNA, which was meaningful for exploring new measures for inhibiting tumor amplification (Li et al., 2013). These findings indicate that berberine derivatives, like berberine, have anti-tumor effects.

### Modification Transformation and Antineoplastic Activity of C-9-Substituted Berberine Derivatives

**Figure 3:**
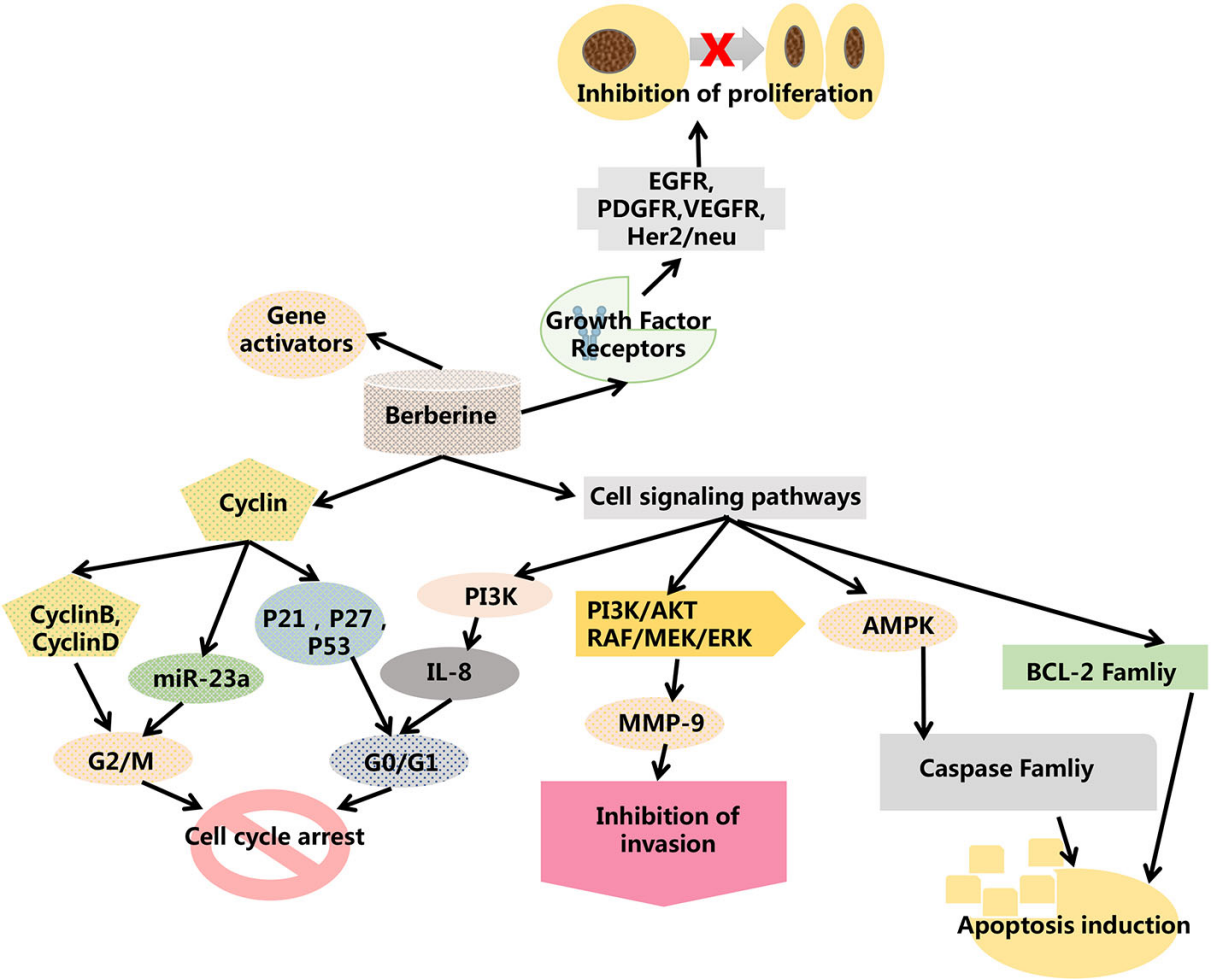
Structure of compounds 15-17.

Shi et al. designed and synthesized a series of new triazole berberine derivatives (Shi et al., 2011). Most of the compounds displayed stronger anti-tumor activity in SMMC-7721 cells than berberine (Lo et al., 2013). Among these derivatives, compounds 12 and 13 exhibited the strongest inhibition activity in SMMC-7721 cell lines. New 1,13-cycloprotoberberine derivatives were designed and synthesized and their cytotoxicity evaluated in HCT 116 (Pang et al., 2005). The results were reported to demonstrate that the replacement of 9-methoxyl with an ester moiety strengthened *in vitro* antiproliferative activity. Further research indicated that compound 14 inhibited the activity of DNA topoisomerase I (Top I), leading to stasis of G2/M phase to decrease the growth of tumor cells. This group also compounded a series of cycloberberine derivatives and evaluated their anticancer activity *in vitro*. The results demonstrated that compounds 15-17 could inhibit human HepG2 cell proliferation induced by the inhibition of DNA Top I at the G2/M phase (**Figure 3**). In addition, they designed and synthesized 9-O-bile acid berberine, which displayed much stronger inhibition on hepatoma carcinoma cells than berberine. This derivative also had higher reported bioavailability (Chen et al., 2005).

Lo’s team observed that lipotropic substitutive derivatives, such as 9-O-alkyl supersede berberine and 9-O-Terpenyl substituted berberine, could induce apoptosis in HepG2 tumor cells and that their inhibitory activity against HepG2 and HT-29 tumor cells could be further enhanced with the extension of the alkyl chain. Moreover, berberine can produce a series of 9-O-berberine derivatives by pyrogenic and alkylation reaction (Mistry et al., 2017). The reaction can convert the methyl in the C (9) methoxy group into ethyl to compound 9-O-ethylamine berberine, demonstrating a strong affinity with CT DNA. Furthermore, compared to the parent berberine, the affinity of berberine red dimer, compounded by Chen’s team (Qin et al., 2006), for DNA increased by 100 times. Protein POT1 is another important protein associated with telomere extension and protection. It has been identified as a potential drug target for cancer treatment. Xiao’s team observed that the interaction between 9-N-supersede berberine derivatives and POT1 could influence the effect of POT1 and telomeric DNA. This result may provide a potential new pathway for future cancer therapies.

Mistry and colleagues combined the C-9 replacement of berberine and substituted 2-aminobenzothiazoles by a pentyl side chain, which was assessed for *in vitro* antioxidant and anticancer activities against tumor cells (Sun et al., 2009; Mistry et al., 2017). The results suggest that compounds with methoxy or cyano functional groups were the most active radical scavengers to DPPH and ABTS. Furthermore, they also exhibited the strongest anti-tumor activity against the HeLa and CaSki cervical cancer cell lines, including the SK-OV-3 ovarian cancer cell line.

Notably, the G-quadruplex in the promoter region of c-myc took effect as a transcriptional repressor. The c-myc transcriptional repressor 1,5-7 controlled by G-quadruplex structure is considered an attractive target for anti-cancer therapeutic strategies. Ma et al. (2008) explored the interaction between 9-N-substituted berberine derivatives and G-quadruplex DNA using electrophoretic mobility shift assay (EMSA), circular dichroism spectroscopy (CD), the fluorescence resonance energy transfer-melting (FRET-melting) method, polymerase chain reaction-stop assay (PCR-stop assay), the competition dialysis method, cell proliferation assay, and reverse transcription-polymerase chain reaction (RT-PCR). The results indicated that these derivatives could selectively induce and stabilize the formation of the c-myc in the parallel molecular G-quadruplex. Accordingly, transcription of c-myc was down-regulated in the cancer cell line. Moreover, this result could be effective with or without metal cations. In addition, the different structural derivatives reported different abilities to stabilize the c-myc G-quadruplex. Therefore, 9-N-substitutes, such as a 1,6-diaminohexyl side chain, at the 9-position of berberine improved the selective binding with G-quadruplex, increased the inhibition of hybridization, and thus blocked gene expression.

Nitric oxide (NO) is also important in various physiological and pathophysiological processes (Chiu and Chien, 2011). Based on recent studies, decreased levels of NO in liver tissues may be conducive to the progression of HCC. In addition, NO donors, such as sodium nitroprusside (SNP), arrested the cell cycle and induced apoptosis in HepG2 cells, suppressing proliferation, migration, and invasion of cancer cells effectively. Therefore, in anti-tumor studies, NO donors are usually used as substitutes for NO. Moreover, numerous reports have demonstrated that the anti-tumor activity of NO-donating hybrids was greater than that of sole NO donors, parent drugs, or their combinations (Bauer et al., 2010; van der Stoep et al., 2009; Wang et al., 2013; Dzinic et al., 2014). Furthermore, NO-donating anti-tumor drugs do not induce any drug resistance in tumor cells. In addition, the hydrophilic nature of the quaternary ammonium salt is the main cause of impaired intestinal absorption. Different lengths of alkyl linkages can be used to improve lipophilicity and enhance effectiveness. Hence, it is necessary to design and synthesize a series of berberine derivatives for the treatment of HCC. Several results have demonstrated that the majority of derivatives with antiproliferative activity against HepG2 cells have been a dramatic improvement on the parent compounds (Nechepurenko et al., 2011). Among these derivatives, compound 15a, superior to the positive control cisplatin, exhibited the most promising activity. In structure-activity relationship analysis, the concentration of released NO increased slightly with the extension of the chain length. A more detailed study indicated that compound 18 resulted in the stasis of the G2 phase in the cell cycle and induced apoptosis in HepG2 cells by the depolarization of the mitochondria. Furthermore, the *in vivo* anti-tumor activity of compound 18 was observed in an H22 liver cancer xenograft mouse model. Therefore, it can be stated that berberine derivatives demonstrate anti-tumor activities (Mei et al., 2015; Zou et al., 2017) and could be promising therapeutic agents in cancer therapy.

## Conclusion

Berberine and its derivatives have extensive pharmacological actions. In this review, their anti-tumor effects and underlying mechanisms were systemically outlined based on systems pharmacology. This review also revealed the efficacy and potential mechanism of action of berberine and its derivatives when used in the treatment of complex cancers at the molecular, cellular, and organism levels. Furthermore, several signaling pathways were also outlined. However, there are limitations in the study of berberine, as its anti-tumor mechanism has yet to be fully elucidated. More importantly, there have only been a few *in vivo* and pre-clinical studies evaluating berberine. However, in-depth investigations on the efficacy of berberine and its derivatives are ongoing, and a growing number of studies have started focusing on the potential anti-tumor role of berberine, possibly mediated through immune regulation. The ability of berberine to increase chemosensitivity and reduce the side effects of chemosensitizers has also been emphasized. Berberine and its derivatives may be promising drugs in cancer therapy, possibly improving clinical efficacy and safety.

## Author Contributions

CZ and LZ wrote the first draft. WY, JS, and XY provided the organization and framework of the article. ZZ, LS, YW, RC, and GL provided critical revisions. All authors approved the final version of the manuscript for submission.

## Funding

This work was supported by National Key R&D Program of China (Grant # 2018YFC1311600), Jilin Science and Technology Agency funding (20180520124JH, 20180519003JH, 20190701078GH and 20180414050GH) and Jilin Province medical and health talents (2019SCZT007).

## Conflict of Interest

The authors declare that the research was conducted in the absence of any commercial or financial relationships that could be construed as a potential conflict of interest.
